# Supplementary material for: Robust group- but limited individual-level (longitudinal) reliability and insights into cross-phases response prediction of conditioned fear
Source: eLife. 2022 Sep 13;11:e78717. doi: 10.7554/eLife.78717 (PMC9691022; doi:10.7554/eLife.78717)
Supplement: Supplementary file 1. [file elife-78717-supp1.docx]

***Supplementary File 1:*** Overview of experimental specifications and results of five previous studies reporting test-retest reliabilities in human fear conditioning research.

|  | **Fredrikson et al., 1993** | **Zeidan et al., 2012** | **Torrents-Rodas et al., 2014** | **Ridderbusch et al., 2021** | **Cooper et al., PREPRINT** |
| --- | --- | --- | --- | --- | --- |
| **N/female/male/age** | 28/14/14/M = 28.5 (± 1.42) | 18/9/9/M = 38.0 (± 12.7) | 71/52/19/M = 22.4 (± 2.61) | 100/46/54/M = 33.1 (± 10.7) | 51/39/12/ M = 20.0 (± 2.88) |
| **Reinforcement rate (%)** | 100 | 100 | Acquisition training: 75 Generalization: 50 | 60 | 40 |
| **Acquisition type** | Not reported | Not reported | Uninstructed but informed about the existence of contingencies^3^ | Instructed (but not informed about the reinforcement rate) | Instructed (but not informed about the reinforcement rate) |
| **Extinction type** | Immediate | Immediate; Extinction training consisted of 2 subphases separated by a 1-min rest period | None | 24h delayed; Extinction training consisted of 2 subphases (Ex1 and Ex2) | None |
| **Additional phase(s)** | None | 24h delayed extinction recall immediately followed by renewal | Generalization (10 min. after acquisition training) | One re-acquisition trial prior to extinction training Reinstatement-test (immediately after extinction training and reinstatement) | Generalization |
| **CS quality** | Geometric shapes | Lamp in a room (2 colors) | 2 rings as CSs and 8 rings as GSs | Neutral faces on colored background (background color = CS type) | Auditory (pure tone sine waves < 60 decibels): CS+ and CS- = 1000 and 550 Hz; 6 GSs = 650, 800, 900, 1100, 1200 and 1350 Hz |
| **CS duration (s)** | 8 | 12 | 8 | 6 | 2.5 |
| **ITI duration (s)** | 20 – 40 | 12 – 21 | 9 – 17 | 6 – 10 | 7 – 8 |
| **US type** | Auditory (110 dB white noise) | Electrotactile | Electrotactile | Electrotactile | Electrotactile |
| **# of habituation trials CS+/CS-** | 4/4 | 4/4 | 6/6 | 2/2 | No habituation phase |
| **# of acquisition trials  CS+/CS-** | 8/8 | 5/5 | 12/12 | 10/10 | 20/12 |
| **# of extinction trials  CS+/CS-** | 8/8 | 5/5 (in each of the 2 subphases) | No extinction phase | Extinction phase 1 (Ex1): 10/10 Extinction phase 2 (Ex2): 10/10 | No extinction phase |
| **# of trials add. phase  CS+/CS-** | No additional phase | Extinction recall: 5/5 Renewal: 5/5 | 12/12 6 times each GS | Re-acquisition: 1/0 Reinstatement-test: 10/10 | Generalization: 12/7 7 times each GS |
| **SCR** | Yes | Yes | Yes | Yes | Yes |
| **FPS** | No | No | Yes | Yes | No |
| **Ratings** | No | No | Risk ratings | Expectancy, arousal, valence ratings | Shock risk ratings |
| **fMRI** | No | No | No | Yes | No |
| **Reported measure(s)** | SCR | SCR | SCR, FPS, ratings | fMRI, ratings | SCR, ratings |
| **# of measurement time points** | 2 | 3 | 2 | 2 | 2 |
| **Time gap between measurement time points** | 20 days | Time points 1 and 2: 17.9 ± 2.1 weeks  Time points 2 and 3: 14.5 ± 0.7 weeks | 5.8 - 9.0 months (M = 7.7) | 13 weeks | 9 days |
| **Same stimuli used in retest** | Not reported | No^1^ | Yes (half of the participants) New set (other half of the participants (new stimuli = lines with varying slopes) | No^5^ | Yes |
| **Same allocation of stimuli to CSs** | Not reported | No^2^ | Yes (applies to the use of the same stimulus set) | No^5^ | Yes |
| **Reliability measure** | Pearson’s r | ICC (no type specified) | G coefficient (range = 0 - 1) ^4^ | ICC(1,1)^6^ | G coefficient (range = 0 - 1) ^4^ |
| **Included trials** | All | All | All | See results and notes below | All |
| **Test-Retest Habituation** |  |  |  |  |  |
| **CS+** | SCR (FIR): 0.62 | SCR (time points 1-3): 0.10 SCR (time points 1-2): 0.16 | Not reported | **fMRI** No fMRI data for habituation  **Ratings** No rating data for habituation | No habituation phase |
| **CS-** | SCR (FIR): 0.72 | Not reported |  |  |  |
| **Test-Retest Acquisition** |  |  |  |  |  |
| **CS+** | SCR (FIR): 0.85 SCR (SIR): 0.51 SCR (TIR): 0.65 | SCR (time points 1-3): 0.68 SCR (time points 1-2): 0.64 | **Same stimulus set**^4^ SCR: 0.27 FPS: 0.34 Ratings: 0.23  **New stimulus set**^4^ SCR: 0.39 FPS: 0.40 Ratings: 0.46 | **fMRI** No fMRI data for acquisition training  **Ratings** Expectancy: not reported Arousal: no data for acquisition training Valence: no data for acquisition training | SCR^4^: 0.50  Ratings^4^: 0.47 |
| **CS-** | SCR (FIR): 0.57 SCR (SIR): 0.27 SCR (TIR): 0.29 | Not reported |  |  |  |
| **CS discrimination** | Not included | SCR (time points 1-3): 0.43 |  |  |  |
| **Test-Retest Extinction** |  |  |  |  |  |
| **CS+** | SCR (FIR): 0.62 SCR (SIR): 0.27 SCR (TIR): 0.83 | SCR (time points 1-3): -0.19 SCR (time points 1-2): -0.24 | No extinction phase | **fMRI Ex1** Right insula: 0.54 Left insula: 0.57 Middle cingulate cortex: 0.40  **Ex1 > Ex2** Left insula: 0.22 Right insula: 0.14 Middle cingulate cortex: 0.29  **Ratings pre Ex1** Expectancy: no data for pre Ex1 Arousal: not reported Valence: not reported  **Post Re-Acq, post Ex1 and post Ex2^7^** Expectancy: 0.66 Arousal: 0.63 Valence: 0.56 | No extinction phase |
| **CS-** | SCR (FIR): 0.37 SCR (SIR): -0.05 SCR (TIR): 0.09 | Not reported |  | Not reported |  |
| **CS discrimination** | Not included | Not reported |  | **fMRI Ex1** Right insula: 0.44 Left insula: 0.39 Middle cingulate cortex: 0.34  **Ex1 > Ex2** Left insula: 0.20 Right insula: 0.01 Middle cingulate cortex: 0.13  **Ratings pre Ex1** Expectancy: no data for pre Ex1 Arousal: 0.42 Valence: 0.02  **Post Re-Acq, post Ex1 and post Ex2^7^** Expectancy: 0.64 Arousal: 0.43 Valence: 0.25 |  |
| **Test-Retest additional phase** |  | **Extinction recall Renewal** | **Generalization** | **Re-Acquisition Reinstatement-Test** | **Generalization** |
| **CS+** | No additional phase | **Extinction recall:** SCR (time points 1-3): 0.46 SCR (time points 1-2): 0.72  **Renewal:** SCR (time points 1-3): 0.67 SCR (time points 1-2): 0.66 | **Same stimulus set**^4^ SCR: 0.44 FPS: 0.22 Ratings: 0.22  **New stimulus set**^4^ SCR: 0.21 FPS: 0.16 Ratings: 0.25 | **fMRI** Not reported  **Ratings post Re-Acquisition** Expectancy: 0.51 Arousal: 0.53 Valence: 0.49 | SCR^4^: 0.39  Ratings^4^: 0.36 |
| **CS-** |  | Not reported |  | Not reported |  |
| **CS discrimination** |  | **Extinction Recall** SCR (time points 1-3): 0.23  **Renewal** SCR (time points 1-3): 0.50 |  | **fMRI RI-T: Cingulate cortex cluster** pre RI^8^: 0.01 post RI^9^: -0.05 pre vs. post RI^8,9^: -0.12  **Ratings post Re-Acquisition** Expectancy: 0.49 Arousal: not reported Valence: not reported  **pre RI**^10^ Expectancy: 0.67 Arousal: 0.53 Valence: 0.39  **post RI** Expectancy: 0.52 Arousal: 0.55 Valence: 0.34  **pre vs. post RI**^10^ Expectancy: 0.22 Arousal: 0.19 Valence: -0.03 |  |
| **Physiological response quantification** |  |  |  |  |  |
| **SCR quantification** | Trough-to-peak (TTP) | Baseline correction | Baseline correction | Trough-to-peak (TTP) | Trough-to-peak (TTP) |
| **SCR scoring criteria** | FIR: 1-4 s after CS onset SIR: 5-9 s after CS onset TIR: 1-4 s after CS termination | Baseline: means SCL during 2 s before trial onset  subtracted from the highest SCL within the 12 s CS duration | Value at stimulus onset subtracted from the maximum value during 1-5 s after stimulus onset (only trials without risk ratings analyzed) | First response occurring 0.9-4 s after stimulus onset | First response occurring 0.5-3 s after stimulus onset, lasting for 0.5 and 5.0 s, > 0.02 µS |
| **FPS specifications** | No FPS applied | No FPS applied | 5s after onset of odd trials and during ITIs (6 times per phase, IPIs 18-25 s) | Either 4.5 or 5 s after CS onset and during ITI (2, 3, 4, 5, or 6 s after CS offset); presented during all CS trials during habituation and during 8 of 10 CS trials during fear acquisition training | No FPS applied |
| **FPS quantification** |  |  | Baseline correction | Trough-to-peak (TTP) |  |
| **FPS scoring criteria** |  |  | Value at response peak (Response onset in a time window 20-100 ms after probe onset with a peak between 20 and 150 ms after probe onset) subtracted from a baseline value (averaged during the 50 ms preceding the probe) | Response in a time window 20-120 ms after probe onset with a maximum peak within 150 ms after onset |  |
| **Ratings provided** | No ratings provided | No ratings provided | During even trials | Expectancy: before each CS trial  Arousal and Valence: post Re-Acq, pre Ex1, post Ex1, post Ex2, post RI, post RIT | Trial-by-trial shock expectancy |

*Note.* We are aware that there is another study by Savage et al (2019) which investigated the test-retest reliability of fear potentiated startle in a differential fear conditioning paradigm. But since the participants of this study were twins and relatively young (age*_M_* = 11.5, age*_SD_* = 1.5), we have not included them in the table due to reduced comparability. # = number; FIR = first interval response, occurring 1-4s after CS onset; SIR = second interval response, occurring 5-9s after CS onset; TIR = third interval response, occurring 1-4s after CS termination; GS = generalization stimulus; Ex1 = first extinction phase; Ex2 = second extinction phase; pre/post = prior and subsequent to respective phases; Re-Acq = re-acquisition; RI-T = reinstatement-test; RI = reinstatement.

^1^ “Conditioning context and color of the CS+ were different for each of the 3 sessions and counterbalanced across visits.” (Zeidan et al., 2012, p. 314)

^2^ “The conditioning context and the color of the CS+ were different for each of the three test sessions and counterbalanced across visits.” (Zeidan et al., 2012, p. 315)

^3^ “They were not instructed about the CS−US contingency, but were told that they might learn to predict the shock if they pay attention to the presented stimuli.” (Torrents-Rodas et al., 2014, p. 699)

^4^ The calculation of the G coefficient includes both responses to the CS+ and CS-. Thus, results are not separated for stimulus types.

^5^ “The whole experimental protocol (t1) was repeated after an interval of an average of 13 weeks (second measurement: t2), using two different visual stimuli as CSs to avoid re ‐acquisition.” (Ridderbusch et al., 2021, p. 3)

^6^ One-way random effects model with single measures.

^7^ Post re-acq, post Ex1 and post Ex2 = “extinction training effect” (see Ridderbusch et al., 2021).

^8^ Pre RI means for fMRI: last half of Ex2 trials (5 trials).

^9^ Post RI means for fMRI: first half of RI-T (5 trials).

^10^ Pre RI means for ratings: post Ex2.

**References**

Cooper, S. E., Dunsmoor, J. E., Koval, K., Pino, E., & Steinman, S. (2022). *Test-Retest Reliability of Human Threat Conditioning and Generalization* [Preprint]. PsyArXiv. <https://doi.org/10.31234/osf.io/84uqz>

Fredrikson, M., Annas, P., Georgiades, A., Hursti, T., & Tersman, Z. (1993). Internal consistency and temporal stability of classically conditioned skin conductance responses. *Biological Psychology*, *35*(2), 153–163. <https://doi.org/10.1016/0301-0511(93)90011-V>

Ridderbusch, I. C., Wroblewski, A., Yang, Y., Richter, J., Hollandt, M., Hamm, A. O., Wittchen, H.-U., Ströhle, A., Arolt, V., Margraf, J., Lueken, U., Herrmann, M. J., Kircher, T., & Straube, B. (2021). Neural adaptation of cingulate and insular activity during delayed fear extinction: A replicable pattern across assessment sites and repeated measurements. *NeuroImage*, *237*, 118157. <https://doi.org/10.1016/j.neuroimage.2021.118157>

Savage, J. E., Moore, A. A., Sawyers, C. K., Bourdon, J. L., Verhulst, B., Carney, D. M., Moroney, E., Machlin, L., Kaabi, O., Vrana, S., Grillon, C., Brotman, M. A., Leibenluft, E., Pine, D. S., Roberson‐Nay, R., & Hettema, J. M. (2019). Fear‐potentiated startle response as an endophenotype: Evaluating metrics and methods for genetic applications. *Psychophysiology*, *56*(5), e13325. <https://doi.org/10.1111/psyp.13325>

Torrents-Rodas, D., Fullana, M. A., Bonillo, A., Andión, O., Molinuevo, B., Caseras, X., & Torrubia, R. (2014). Testing the temporal stability of individual differences in the acquisition and generalization of fear: Stability acquisition and generalization of fear. *Psychophysiology*, *51*(7), 697–705. <https://doi.org/10.1111/psyp.12213>

Zeidan, M. A., Lebron‐Milad, K., Thompson‐Hollands, J., Im, J. J. Y., Dougherty, D. D., Holt, D. J., Orr, S. P., & Milad, M. R. (2012). Test–Retest Reliability during Fear Acquisition and Fear Extinction in Humans. *CNS Neuroscience & Therapeutics*, *18*(4), 313–317. <https://doi.org/10.1111/j.1755-5949.2011.00238.x>
