# Supplementary material for: Robust group- but limited individual-level (longitudinal) reliability and insights into cross-phases response prediction of conditioned fear
Source: eLife. 2022 Sep 13;11:e78717. doi: 10.7554/eLife.78717 (PMC9691022; doi:10.7554/eLife.78717)
Supplement: Supplementary file 2. [file elife-78717-supp2.docx]

***Supplementary File 2:*** Deviations from pre-registration.

| **Pre-registration** | **Deviation type** | **Manuscript** | **Justification** |
| --- | --- | --- | --- |
| Not pre-registered | Additional analyses | Analyses of cross-sectional reliability | Considered to provide additional valuable information |
| Mixed-effects approach for calculation of ICCS | Changes analysis approach | ANOVA approach for calculation of ICCS | Statistical approach changed due to model non-convergence problems |
| Calculate ICCs for ranked and non-ranked data | Omitted pre-registered specification | Non-ranked ICCs only | During closer inspection of the conceptualization of ICC_con_, we realised that it would be redundant to calculate both ICC_abs_ and ICC_con_ with ranked and non-ranked data as ICC_con_ itself ranks the data. Hence, we decided to calculate ICCs based on non-ranked data only. |
| Not pre-registered | Additional analyses | Inclusion of ICC for SCRs to the US and US aversiveness ratings | Considered to provide valuable information |
| Not pre-registered | Additional analyses | Additional phase operationalization: last two extinction trials | Considered to provide valuable information for completeness |
| Not pre-registered | Additional analyses | Trial-by trial ICCs for SCRs | Considered to provide additional valuable information |
| Not pre-registered | Additional analyses | Inclusion of analysis focusing on reliability at the group level for SCRs | Considered to provide additional valuable information |
| Not pre-registered | Additional outcome measure | Inclusion of fMRI as an outcome measure and corresponding reliability analyses as well as within-session predictability analyses | Considered to provide valuable information |
| Multiple linear regression with SCRs or fear ratings during both acquisition and extinction training as multiple predictors for responses at reinstatement-test | Changes in analysis approach | Simple linear regressions including SCRs or fear ratings during acquisition training as predictors and responding during reinstatement-test as criterion. Further checks of statistical assumptions revealed heteroscedasticity of the data. Therefore, we conducted simple linear regressions with robust standard errors instead of using classical OLS estimators | Due to multicollinearity of the predictors resulting from significant associations of responding during acquisition and extinction training the pre-registered analyses were not suitable |
| Not pre-registered | Additional analyses | We compare different patterns of SCR, fear rating and fMRI data after pooling them for certain data specifications/ROIs in predictability analyses | Considered to provide additional valuable information |
