# Supplementary material for: Robust group- but limited individual-level (longitudinal) reliability and insights into cross-phases response prediction of conditioned fear
Source: eLife. 2022 Sep 13;11:e78717. doi: 10.7554/eLife.78717 (PMC9691022; doi:10.7554/eLife.78717)
Supplement: Supplementary file 3. [file elife-78717-supp3.docx]

**Supplementary File 3:** ICC_abs_ and ICC_con_ for all data specifications of SCRs.

| **Outcome** | **Ampl.-type** | **Stim.-type** | **Phase** | **Op.** | **ICC_abs_** | | | | **ICC_con_** | | | |
| --- | --- | --- | --- | --- | --- | --- | --- | --- | --- | --- | --- | --- |
|  |  |  |  |  | **Value** | **Lower 95% CI** | **Upper 95% CI** | **p-value** | **Value** | **Lower 95% CI** | **Upper 95% CI** | **p-value** |
| SCR | raw | CS dis. | Acq | average | 0.160 | -0.020 | 0.340 | .077 | 0.170 | -0.030 | 0.350 | .077 |
| SCR | raw | CS+ | Acq | average | 0.270 | 0.090 | 0.440 | .006 | 0.300 | 0.110 | 0.460 | .006 |
| SCR | raw | CS- | Acq | average | 0.390 | 0.210 | 0.540 | .000 | 0.410 | 0.230 | 0.560 | .000 |
| SCR | raw | US | Acq | average | 0.317 | 0.133 | 0.481 | .003 | 0.322 | 0.135 | 0.487 | .003 |
| SCR | raw | CS dis. | Acq | last 2 trials | 0.240 | 0.060 | 0.420 | .017 | 0.250 | 0.060 | 0.430 | .017 |
| SCR | raw | CS+ | Acq | last 2 trials | 0.220 | 0.040 | 0.390 | .025 | 0.230 | 0.040 | 0.410 | .025 |
| SCR | raw | CS- | Acq | last 2 trials | 0.190 | 0.000 | 0.370 | .055 | 0.190 | -0.010 | 0.370 | .055 |
| SCR | raw | CS dis. | Ext | 1st trial | 0.190 | 0.010 | 0.360 | .044 | 0.200 | 0.010 | 0.380 | .044 |
| SCR | raw | CS+ | Ext | 1st trial | 0.320 | 0.140 | 0.490 | .003 | 0.330 | 0.140 | 0.490 | .003 |
| SCR | raw | CS- | Ext | 1st trial | 0.040 | -0.120 | 0.210 | .331 | 0.050 | -0.140 | 0.250 | .331 |
| SCR | raw | CS dis. | Ext | average | 0.070 | -0.130 | 0.270 | .272 | 0.070 | -0.120 | 0.260 | .272 |
| SCR | raw | CS+ | Ext | average | 0.480 | 0.320 | 0.620 | .000 | 0.500 | 0.340 | 0.630 | .000 |
| SCR | raw | CS- | Ext | average | 0.580 | 0.430 | 0.700 | .000 | 0.610 | 0.470 | 0.720 | .000 |
| SCR | raw | CS dis. | Ext | last 2 trials | 0.060 | -0.140 | 0.250 | .321 | 0.060 | -0.140 | 0.250 | .321 |
| SCR | raw | CS+ | Ext | last 2 trials | 0.170 | -0.020 | 0.360 | .073 | 0.170 | -0.020 | 0.360 | .073 |
| SCR | raw | CS- | Ext | last 2 trials | 0.200 | 0.000 | 0.380 | .048 | 0.200 | 0.000 | 0.380 | .048 |
| SCR | raw | CS dis. | RI-T | 1st trial | 0.140 | -0.050 | 0.320 | .119 | 0.140 | -0.060 | 0.330 | .119 |
| SCR | raw | CS+ | RI-T | 1st trial | 0.150 | -0.040 | 0.330 | .098 | 0.150 | -0.040 | 0.340 | .098 |
| SCR | raw | CS- | RI-T | 1st trial | 0.030 | -0.130 | 0.200 | .389 | 0.030 | -0.160 | 0.230 | .389 |
| SCR | raw | US | RI | average | 0.271 | 0.085 | 0.440 | .009 | 0.278 | 0.087 | 0.449 | .009 |
| SCR | log | CS dis. | Acq | average | 0.180 | 0.000 | 0.350 | .054 | 0.190 | 0.000 | 0.370 | .054 |
| SCR | log | CS+ | Acq | average | 0.290 | 0.100 | 0.450 | .004 | 0.310 | 0.130 | 0.480 | .004 |
| SCR | log | CS- | Acq | average | 0.400 | 0.230 | 0.550 | .000 | 0.420 | 0.250 | 0.570 | .000 |
| SCR | log | US | Acq | average | 0.320 | 0.137 | 0.482 | .003 | 0.327 | 0.140 | 0.491 | .003 |
| SCR | log | CS dis. | Acq | last 2 trials | 0.230 | 0.040 | 0.400 | .024 | 0.230 | 0.040 | 0.410 | .024 |
| SCR | log | CS+ | Acq | last 2 trials | 0.210 | 0.020 | 0.380 | .032 | 0.220 | 0.030 | 0.400 | .032 |
| SCR | log | CS- | Acq | last 2 trials | 0.190 | 0.000 | 0.370 | .052 | 0.190 | 0.000 | 0.370 | .052 |
| SCR | log | CS dis. | Ext | 1st trial | 0.180 | 0.000 | 0.350 | .052 | 0.190 | 0.000 | 0.370 | .052 |
| SCR | log | CS+ | Ext | 1st trial | 0.310 | 0.120 | 0.470 | .004 | 0.310 | 0.120 | 0.480 | .004 |
| SCR | log | CS- | Ext | 1st trial | 0.030 | -0.130 | 0.200 | .368 | 0.040 | -0.160 | 0.230 | .368 |
| SCR | log | CS dis. | Ext | average | 0.060 | -0.140 | 0.260 | .301 | 0.060 | -0.130 | 0.250 | .301 |
| SCR | log | CS+ | Ext | average | 0.490 | 0.320 | 0.620 | .000 | 0.500 | 0.340 | 0.640 | .000 |
| SCR | log | CS- | Ext | average | 0.590 | 0.430 | 0.710 | .000 | 0.620 | 0.480 | 0.720 | .000 |
| SCR | log | CS dis. | Ext | last 2 trials | 0.070 | -0.130 | 0.260 | .283 | 0.070 | -0.130 | 0.260 | .283 |
| SCR | log | CS+ | Ext | last 2 trials | 0.200 | 0.010 | 0.380 | .044 | 0.200 | 0.010 | 0.380 | .044 |
| SCR | log | CS- | Ext | last 2 trials | 0.230 | 0.040 | 0.410 | .027 | 0.230 | 0.030 | 0.410 | .027 |
| SCR | log | CS dis. | RI-T | 1st trial | 0.170 | -0.020 | 0.350 | .076 | 0.170 | -0.030 | 0.350 | .076 |
| SCR | log | CS+ | RI-T | 1st trial | 0.160 | -0.030 | 0.340 | .083 | 0.160 | -0.030 | 0.350 | .083 |
| SCR | log | CS- | RI-T | 1st trial | 0.040 | -0.120 | 0.210 | .337 | 0.050 | -0.150 | 0.240 | .337 |
| SCR | log | US | RI | average | 0.299 | 0.116 | 0.464 | .004 | 0.308 | 0.120 | 0.475 | .004 |
| SCR | log rc | CS dis. | Acq | average | 0.230 | 0.050 | 0.410 | .018 | 0.250 | 0.050 | 0.420 | .018 |
| SCR | log rc | CS+ | Acq | average | 0.490 | 0.310 | 0.630 | .000 | 0.530 | 0.370 | 0.660 | .000 |
| SCR | log rc | CS- | Acq | average | 0.610 | 0.470 | 0.730 | .000 | 0.630 | 0.500 | 0.740 | .000 |
| SCR | log rc | US | Acq | average | 0.112 | -0.086 | 0.301 | .176 | 0.111 | -0.086 | 0.300 | .176 |
| SCR | log rc | CS dis. | Acq | last 2 trials | 0.050 | -0.150 | 0.240 | .337 | 0.050 | -0.150 | 0.240 | .337 |
| SCR | log rc | CS+ | Acq | last 2 trials | 0.300 | 0.120 | 0.470 | .004 | 0.310 | 0.120 | 0.480 | .004 |
| SCR | log rc | CS- | Acq | last 2 trials | 0.190 | -0.010 | 0.370 | .056 | 0.190 | -0.010 | 0.370 | .056 |
| SCR | log rc | CS dis. | Ext | 1st trial | 0.200 | 0.020 | 0.370 | .033 | 0.220 | 0.020 | 0.400 | .033 |
| SCR | log rc | CS+ | Ext | 1st trial | 0.270 | 0.080 | 0.440 | .012 | 0.270 | 0.070 | 0.440 | .012 |
| SCR | log rc | CS- | Ext | 1st trial | -0.090 | -0.250 | 0.090 | .804 | -0.100 | -0.290 | 0.090 | .804 |
| SCR | log rc | CS dis. | Ext | average | 0.350 | 0.160 | 0.510 | .002 | 0.340 | 0.160 | 0.510 | .002 |
| SCR | log rc | CS+ | Ext | average | 0.540 | 0.380 | 0.670 | .000 | 0.560 | 0.410 | 0.680 | .000 |
| SCR | log rc | CS- | Ext | average | 0.620 | 0.440 | 0.740 | .000 | 0.660 | 0.530 | 0.760 | .000 |
| SCR | log rc | CS dis. | Ext | last 2 trials | 0.210 | 0.020 | 0.390 | .037 | 0.210 | 0.020 | 0.390 | .037 |
| SCR | log rc | CS+ | Ext | last 2 trials | 0.360 | 0.170 | 0.520 | .001 | 0.350 | 0.170 | 0.510 | .001 |
| SCR | log rc | CS- | Ext | last 2 trials | 0.440 | 0.270 | 0.590 | .000 | 0.440 | 0.270 | 0.590 | .000 |
| SCR | log rc | CS dis. | RI-T | 1st trial | 0.230 | 0.040 | 0.410 | .023 | 0.240 | 0.040 | 0.410 | .023 |
| SCR | log rc | CS+ | RI-T | 1st trial | 0.170 | -0.020 | 0.360 | .071 | 0.170 | -0.020 | 0.360 | .071 |
| SCR | log rc | CS- | RI-T | 1st trial | 0.150 | -0.030 | 0.330 | .086 | 0.160 | -0.030 | 0.350 | .086 |
| SCR | log rc | US | RI | average | 0.093 | -0.106 | 0.284 | .221 | 0.092 | -0.105 | 0.282 | .221 |
| *Note*. Ampl. = Amplitude, Stim. = Stimulus, Op. = Operationalization, CI = Confidence Interval, CS dis. = CS discrimination, log = log-transformed, log rc = log-transformed and range corrected, Acq = Acquisition training, Ext = Extinction training, RI = Reinstatement, RI-T = Reinstatement-Test. | | | | | | | | | | | | |
