# Supplementary material for: Robust group- but limited individual-level (longitudinal) reliability and insights into cross-phases response prediction of conditioned fear
Source: eLife. 2022 Sep 13;11:e78717. doi: 10.7554/eLife.78717 (PMC9691022; doi:10.7554/eLife.78717)
Supplement: Supplementary file 4. [file elife-78717-supp4.docx]

**Supplementary File 4:** ICC_abs_ and ICC_con_ for all data specifications of fear ratings.

| **Outcome** | **Stim.-type** | **Phase** | **Op.** | **ICC_abs_** | | | | **ICC_con_** | | | |
| --- | --- | --- | --- | --- | --- | --- | --- | --- | --- | --- | --- |
|  |  |  |  | **Value** | **Lower 95% CI** | **Upper 95% CI** | **p-value** | **Value** | **Lower 95% CI** | **Upper 95% CI** | **p-value** |
| Ratings | CS dis. | Acq | post-pre | 0.190 | 0.007 | 0.364 | .043 | 0.203 | 0.008 | 0.384 | .043 |
| Ratings | CS+ | Acq | post-pre | 0.436 | 0.262 | 0.582 | < .001 | 0.433 | 0.260 | 0.579 | < .001 |
| Ratings | CS- | Acq | post-pre | -0.162 | -0.328 | 0.018 | .945 | -0.190 | -0.372 | 0.005 | .945 |
| Ratings | CS dis. | Acq | post | 0.424 | 0.230 | 0.581 | < .001 | 0.470 | 0.302 | 0.609 | < .001 |
| Ratings | CS+ | Acq | post | 0.343 | 0.163 | 0.502 | .001 | 0.362 | 0.179 | 0.521 | .001 |
| Ratings | CS- | Acq | post | 0.228 | 0.045 | 0.400 | .020 | 0.242 | 0.049 | 0.417 | .020 |
| Ratings | US | Acq | post | 0.310 | 0.120 | 0.470 | .005 | 0.300 | 0.110 | 0.470 | .005 |
| Ratings | CS dis. | Ext | pre | 0.459 | 0.250 | 0.617 | < .001 | 0.516 | 0.357 | 0.646 | < .001 |
| Ratings | CS+ | Ext | pre | 0.485 | 0.266 | 0.643 | < .001 | 0.548 | 0.395 | 0.671 | < .001 |
| Ratings | CS- | Ext | pre | 0.702 | 0.587 | 0.789 | < .001 | 0.700 | 0.585 | 0.788 | < .001 |
| Ratings | CS dis. | Ext | pre-post | 0.482 | 0.308 | 0.623 | < .001 | 0.512 | 0.352 | 0.643 | < .001 |
| Ratings | CS+ | Ext | pre-post | 0.494 | 0.282 | 0.648 | < .001 | 0.552 | 0.400 | 0.675 | < .001 |
| Ratings | CS- | Ext | pre-post | 0.188 | 0.003 | 0.363 | .048 | 0.198 | 0.002 | 0.378 | .048 |
| Ratings | CS dis. | Ext | post | 0.165 | -0.025 | 0.345 | .078 | 0.169 | -0.027 | 0.353 | .078 |
| Ratings | CS+ | Ext | post | 0.474 | 0.307 | 0.613 | < .001 | 0.472 | 0.305 | 0.611 | < .001 |
| Ratings | CS- | Ext | post | 0.686 | 0.563 | 0.779 | < .001 | 0.700 | 0.584 | 0.787 | < .001 |
| Ratings | US | RI | post | 0.430 | 0.260 | 0.580 | < .001 | 0.450 | 0.280 | 0.590 | < .001 |
| Ratings | CS dis. | RI-T | pre | 0.172 | -0.021 | 0.354 | .072 | 0.174 | -0.022 | 0.357 | .072 |
| Ratings | CS+ | RI-T | pre | 0.437 | 0.264 | 0.582 | < .001 | 0.436 | 0.263 | 0.582 | < .001 |
| Ratings | CS- | RI-T | pre | 0.538 | 0.382 | 0.663 | < .001 | 0.536 | 0.381 | 0.662 | < .001 |
| *Note*. Stim. = Stimulus, Op. = Operationalization, CI = Confidence Interval, CS dis. = CS discrimination, Acq = Acquisition training, Ext = Extinction training, RI = Reinstatement, RI-T = Reinstatement-Test, pre = prior to the experimental phase, post = subsequent to the experimental phase. | | | | | | | | | | | |
