# Supplementary material for: Robust group- but limited individual-level (longitudinal) reliability and insights into cross-phases response prediction of conditioned fear
Source: eLife. 2022 Sep 13;11:e78717. doi: 10.7554/eLife.78717 (PMC9691022; doi:10.7554/eLife.78717)
Supplement: Supplementary file 5. [file elife-78717-supp5.docx]

**Supplementary File 5:** ICC_abs_ and ICC_con_ for CS discrimination during fear acquisition (Acq) and extinction training (Ext).

| **Phase** | **ICC-type** | **Whole Brain** | **Anterior Insula** | **Amygdala** | **Hippocampus** | **Caudate Nucleus** | **Putamen** | **Pallidum** | **NAcc** | **Thalamus** | **dACC** | **dlPFC** | **vmPFC** |
| --- | --- | --- | --- | --- | --- | --- | --- | --- | --- | --- | --- | --- | --- |
| **Acq** | ICCabs | 0.175 | 0.001 | < 0.001 | < 0.001 | < 0.001 | < 0.001 | < 0.001 | < 0.001 | < 0.001 | < 0.001 | < 0.001 | 0.001 |
|  | ICCcon | 0.175 | 0.001 | < 0.001 | < 0.001 | < 0.001 | < 0.001 | < 0.001 | < 0.001 | < 0.001 | < 0.001 | < 0.001 | 0.001 |
| **Ext** | ICCabs | 0.008 | < 0.001 | < 0.001 | < 0.001 | < 0.001 | < 0.001 | < 0.001 | < 0.001 | < 0.001 | < 0.001 | < 0.001 | < 0.001 |
|  | ICCcon | 0.008 | < 0.001 | < 0.001 | < 0.001 | < 0.001 | < 0.001 | < 0.001 | < 0.001 | < 0.001 | < 0.001 | < 0.001 | < 0.001 |
| *Note*. NAcc = nucleus accumbens; dACC = dorsal anterior cingulate cortex; dlPFC = dorsolateral prefrontal cortex; vmPFC = ventromedial prefrontal cortex. | | | | | | | | | | | | | |
