# Supplementary material for: Robust group- but limited individual-level (longitudinal) reliability and insights into cross-phases response prediction of conditioned fear
Source: eLife. 2022 Sep 13;11:e78717. doi: 10.7554/eLife.78717 (PMC9691022; doi:10.7554/eLife.78717)
Supplement: Supplementary file 6. [file elife-78717-supp6.docx]

**Supplementary File 6:** Paired sample t-tests comparing between- and within-subject similarity for whole brain activation pattern as well as activation pattern in the ROIs for acquisition training (Acq) and extinction training (Ext).

| **Phase** | **ROI** | ***t*** | ***df*** | ***p*** | ***Cohen's d*** |
| --- | --- | --- | --- | --- | --- |
| **Acq** | Whole Brain | 4.09 | 70 | **< .001** | 0.49 |
|  | Anterior Insula | 4.33 | 70 | **< .001** | 0.51 |
|  | Amygdala | 2.01 | 70 | **.048** | 0.24 |
|  | Hippocampus | 2.18 | 70 | **.033** | 0.26 |
|  | Caudate Nucleus | 2.27 | 70 | **.026** | 0.27 |
|  | Putamen | 2.42 | 70 | **.018** | 0.29 |
|  | Pallidum | 1.84 | 70 | .070 | 0.22 |
|  | NAcc | -0.18 | 70 | .857 | -0.02 |
|  | Thalamus | 3.20 | 70 | **.002** | 0.38 |
|  | dACC | 3.75 | 70 | **< .001** | 0.44 |
|  | dlPFC | 4.71 | 70 | **< .001** | 0.56 |
|  | vmPFC | 2.39 | 70 | **.019** | 0.28 |
| **Ext** | Whole Brain | 1.44 | 70 | .154 | 0.17 |
|  | Anterior Insula | 0.63 | 70 | .531 | 0.07 |
|  | Amygdala | -0.35 | 70 | .726 | -0.04 |
|  | Hippocampus | 0.89 | 70 | .379 | 0.11 |
|  | Caudate Nucleus | -0.65 | 70 | .520 | -0.08 |
|  | Putamen | -0.63 | 70 | .528 | -0.08 |
|  | Pallidum | 0.34 | 70 | .733 | 0.04 |
|  | NAcc | 1.03 | 70 | .306 | 0.12 |
|  | Thalamus | -0.84 | 70 | .401 | -0.10 |
|  | dACC | 0.05 | 70 | .956 | 0.01 |
|  | dlPFC | -0.39 | 70 | .697 | -0.05 |
|  | vmPFC | -0.06 | 70 | .955 | -0.01 |
| *Note*. NAcc = nucleus accumbens; dACC = dorsal anterior cingulate cortex; dlPFC = dorsolateral prefrontal cortex; vmPFC = ventromedial prefrontal cortex. | | | | | |
