# Supplementary material for: Robust group- but limited individual-level (longitudinal) reliability and insights into cross-phases response prediction of conditioned fear
Source: eLife. 2022 Sep 13;11:e78717. doi: 10.7554/eLife.78717 (PMC9691022; doi:10.7554/eLife.78717)
Supplement: Supplementary file 7. [file elife-78717-supp7.docx]

# Detailed results of predictability analysis: SCR

Cohen’s f^2^ (formula: f^2^ = R^2^/1 – R^2^) was calculated as effect size. According to the guidelines of Cohen (1988), f^2^ $\geq$ .02, f^2^ $\geq$ .15 and f^2^ $\geq$ .34 represent small, medium and large effect sizes respectively. Since Cohen’s f^2^ is informative, but less common (Selya, Rose, Dierker, Hedeker, & Mermelstein, 2012), additionally R squared is reported as effect size.

**Supplementary File 7:** Detailed results of linear regressions: SCR.

| **Outcome** | **Stim.-type** | **Ampl.-type** | **Ranking** | **Predictor** | **Criterion** | ***b*** | ***SE_b_*** | **Lower 95% CI** | **Upper 95% CI** | ***t*** | ***df*** | ***p*** | ***R^2^*** | ***Cohen's f^2^*** |
| --- | --- | --- | --- | --- | --- | --- | --- | --- | --- | --- | --- | --- | --- | --- |
| SCR | CS dis. | raw | not ranked | AVE ACQ | 1st trial EXT | 0.329 | 0.129 | 0.076 | 0.582 | 2.543 | 105 | 0.012 | 0.038 | 0.040 |
| SCR | CS dis. | raw | not ranked | AVE last 2 trials ACQ | 1st trial EXT | 0.264 | 0.080 | 0.107 | 0.421 | 3.288 | 105 | 0.001 | 0.066 | 0.071 |
| SCR | CS dis. | raw | not ranked | AVE ACQ | AVE EXT | 0.109 | 0.062 | -0.013 | 0.231 | 1.762 | 105 | 0.081 | 0.050 | 0.052 |
| SCR | CS dis. | raw | not ranked | AVE last 2 trials ACQ | AVE EXT | 0.031 | 0.031 | -0.030 | 0.092 | 0.986 | 105 | 0.327 | 0.011 | 0.011 |
| SCR | CS dis. | raw | not ranked | AVE ACQ | AVE last 2 trials EXT | 0.081 | 0.115 | -0.144 | 0.306 | 0.705 | 105 | 0.483 | 0.007 | 0.007 |
| SCR | CS dis. | raw | not ranked | AVE last 2 trials ACQ | AVE last 2 trials EXT | -0.039 | 0.105 | -0.245 | 0.167 | -0.371 | 105 | 0.711 | 0.005 | 0.005 |
| SCR | CS dis. | raw | not ranked | AVE ACQ | 1st trial RI-Test | 0.195 | 0.276 | -0.346 | 0.736 | 0.708 | 105 | 0.480 | 0.008 | 0.008 |
| SCR | CS dis. | raw | not ranked | AVE last 2 trials ACQ | 1st trial RI-Test | 0.218 | 0.230 | -0.233 | 0.669 | 0.945 | 105 | 0.347 | 0.028 | 0.029 |
| SCR | CS dis. | raw | not ranked | 1st trial EXT | 1st trial RI-Test | 0.038 | 0.165 | -0.285 | 0.361 | 0.231 | 105 | 0.817 | 0.001 | 0.001 |
| SCR | CS dis. | raw | not ranked | AVE EXT | 1st trial RI-Test | 0.222 | 0.501 | -0.760 | 1.204 | 0.443 | 105 | 0.659 | 0.003 | 0.003 |
| SCR | CS dis. | raw | not ranked | AVE last 2 trials EXT | 1st trial RI-Test | -0.316 | 0.824 | -1.931 | 1.299 | -0.384 | 105 | 0.702 | 0.020 | 0.020 |
| SCR | CS+ | raw | not ranked | AVE ACQ | 1st trial EXT | 0.686 | 0.128 | 0.435 | 0.937 | 5.347 | 105 | 0.000 | 0.291 | 0.410 |
| SCR | CS+ | raw | not ranked | AVE last 2 trials ACQ | 1st trial EXT | 0.508 | 0.130 | 0.253 | 0.763 | 3.909 | 105 | 0.000 | 0.212 | 0.269 |
| SCR | CS+ | raw | not ranked | AVE ACQ | AVE EXT | 0.283 | 0.076 | 0.134 | 0.432 | 3.705 | 105 | 0.000 | 0.273 | 0.375 |
| SCR | CS+ | raw | not ranked | AVE last 2 trials ACQ | AVE EXT | 0.216 | 0.077 | 0.065 | 0.367 | 2.827 | 105 | 0.006 | 0.212 | 0.270 |
| SCR | CS+ | raw | not ranked | AVE ACQ | AVE last 2 trials EXT | 0.200 | 0.099 | 0.006 | 0.394 | 2.006 | 105 | 0.047 | 0.120 | 0.137 |
| SCR | CS+ | raw | not ranked | AVE last 2 trials ACQ | AVE last 2 trials EXT | 0.143 | 0.092 | -0.037 | 0.323 | 1.550 | 105 | 0.124 | 0.082 | 0.089 |
| SCR | CS+ | raw | not ranked | AVE ACQ | 1st trial RI-Test | 0.676 | 0.132 | 0.417 | 0.935 | 5.099 | 105 | 0.000 | 0.213 | 0.270 |
| SCR | CS+ | raw | not ranked | AVE last 2 trials ACQ | 1st trial RI-Test | 0.434 | 0.147 | 0.146 | 0.722 | 2.956 | 105 | 0.004 | 0.117 | 0.132 |
| SCR | CS+ | raw | not ranked | 1st trial EXT | 1st trial RI-Test | 0.608 | 0.101 | 0.410 | 0.806 | 5.993 | 105 | 0.000 | 0.279 | 0.386 |
| SCR | CS+ | raw | not ranked | AVE EXT | 1st trial RI-Test | 1.123 | 0.250 | 0.633 | 1.613 | 4.486 | 105 | 0.000 | 0.172 | 0.208 |
| SCR | CS+ | raw | not ranked | AVE last 2 trials EXT | 1st trial RI-Test | 0.386 | 0.238 | -0.080 | 0.852 | 1.627 | 105 | 0.107 | 0.023 | 0.024 |
| SCR | CS- | raw | not ranked | AVE ACQ | 1st trial EXT | 0.728 | 0.193 | 0.350 | 1.106 | 3.774 | 105 | 0.000 | 0.132 | 0.152 |
| SCR | CS- | raw | not ranked | AVE last 2 trials ACQ | 1st trial EXT | 0.520 | 0.150 | 0.226 | 0.814 | 3.469 | 105 | 0.001 | 0.086 | 0.094 |
| SCR | CS- | raw | not ranked | AVE ACQ | AVE EXT | 0.369 | 0.082 | 0.208 | 0.530 | 4.518 | 105 | 0.000 | 0.280 | 0.390 |
| SCR | CS- | raw | not ranked | AVE last 2 trials ACQ | AVE EXT | 0.231 | 0.074 | 0.086 | 0.376 | 3.126 | 105 | 0.002 | 0.140 | 0.163 |
| SCR | CS- | raw | not ranked | AVE ACQ | AVE last 2 trials EXT | 0.370 | 0.148 | 0.080 | 0.660 | 2.495 | 105 | 0.014 | 0.165 | 0.197 |
| SCR | CS- | raw | not ranked | AVE last 2 trials ACQ | AVE last 2 trials EXT | 0.265 | 0.107 | 0.055 | 0.475 | 2.475 | 105 | 0.015 | 0.107 | 0.120 |
| SCR | CS- | raw | not ranked | AVE ACQ | 1st trial RI-Test | 0.640 | 0.240 | 0.170 | 1.110 | 2.661 | 105 | 0.009 | 0.086 | 0.094 |
| SCR | CS- | raw | not ranked | AVE last 2 trials ACQ | 1st trial RI-Test | 0.449 | 0.239 | -0.019 | 0.917 | 1.878 | 105 | 0.063 | 0.054 | 0.057 |
| SCR | CS- | raw | not ranked | 1st trial EXT | 1st trial RI-Test | 0.336 | 0.118 | 0.105 | 0.567 | 2.849 | 105 | 0.005 | 0.096 | 0.106 |
| SCR | CS- | raw | not ranked | AVE EXT | 1st trial RI-Test | 0.584 | 0.299 | -0.002 | 1.170 | 1.953 | 105 | 0.054 | 0.035 | 0.036 |
| SCR | CS- | raw | not ranked | AVE last 2 trials EXT | 1st trial RI-Test | 0.145 | 0.319 | -0.480 | 0.770 | 0.453 | 105 | 0.651 | 0.004 | 0.004 |
| SCR | CS dis. | log | not ranked | AVE ACQ | 1st trial EXT | 0.328 | 0.134 | 0.065 | 0.591 | 2.446 | 105 | 0.016 | 0.037 | 0.039 |
| SCR | CS dis. | log | not ranked | AVE last 2 trials ACQ | 1st trial EXT | 0.260 | 0.085 | 0.093 | 0.427 | 3.045 | 105 | 0.003 | 0.062 | 0.066 |
| SCR | CS dis. | log | not ranked | AVE ACQ | AVE EXT | 0.109 | 0.056 | -0.001 | 0.219 | 1.956 | 105 | 0.053 | 0.047 | 0.050 |
| SCR | CS dis. | log | not ranked | AVE last 2 trials ACQ | AVE EXT | 0.031 | 0.031 | -0.030 | 0.092 | 1.000 | 105 | 0.320 | 0.010 | 0.010 |
| SCR | CS dis. | log | not ranked | AVE ACQ | AVE last 2 trials EXT | 0.074 | 0.103 | -0.128 | 0.276 | 0.719 | 105 | 0.474 | 0.006 | 0.006 |
| SCR | CS dis. | log | not ranked | AVE last 2 trials ACQ | AVE last 2 trials EXT | -0.039 | 0.105 | -0.245 | 0.167 | -0.373 | 105 | 0.710 | 0.004 | 0.004 |
| SCR | CS dis. | log | not ranked | AVE ACQ | 1st trial RI-Test | 0.135 | 0.259 | -0.373 | 0.643 | 0.521 | 105 | 0.603 | 0.004 | 0.004 |
| SCR | CS dis. | log | not ranked | AVE last 2 trials ACQ | 1st trial RI-Test | 0.173 | 0.221 | -0.260 | 0.606 | 0.784 | 105 | 0.435 | 0.018 | 0.018 |
| SCR | CS dis. | log | not ranked | 1st trial EXT | 1st trial RI-Test | 0.043 | 0.149 | -0.249 | 0.335 | 0.291 | 105 | 0.771 | 0.001 | 0.001 |
| SCR | CS dis. | log | not ranked | AVE EXT | 1st trial RI-Test | 0.149 | 0.450 | -0.733 | 1.031 | 0.331 | 105 | 0.741 | 0.001 | 0.001 |
| SCR | CS dis. | log | not ranked | AVE last 2 trials EXT | 1st trial RI-Test | -0.282 | 0.685 | -1.625 | 1.061 | -0.412 | 105 | 0.681 | 0.017 | 0.017 |
| SCR | CS+ | log | not ranked | AVE ACQ | 1st trial EXT | 0.679 | 0.115 | 0.454 | 0.904 | 5.906 | 105 | 0.000 | 0.297 | 0.423 |
| SCR | CS+ | log | not ranked | AVE last 2 trials ACQ | 1st trial EXT | 0.502 | 0.117 | 0.273 | 0.731 | 4.305 | 105 | 0.000 | 0.210 | 0.265 |
| SCR | CS+ | log | not ranked | AVE ACQ | AVE EXT | 0.294 | 0.074 | 0.149 | 0.439 | 3.995 | 105 | 0.000 | 0.277 | 0.383 |
| SCR | CS+ | log | not ranked | AVE last 2 trials ACQ | AVE EXT | 0.232 | 0.074 | 0.087 | 0.377 | 3.145 | 105 | 0.002 | 0.223 | 0.287 |
| SCR | CS+ | log | not ranked | AVE ACQ | AVE last 2 trials EXT | 0.202 | 0.094 | 0.018 | 0.386 | 2.158 | 105 | 0.033 | 0.117 | 0.133 |
| SCR | CS+ | log | not ranked | AVE last 2 trials ACQ | AVE last 2 trials EXT | 0.149 | 0.088 | -0.023 | 0.321 | 1.686 | 105 | 0.095 | 0.082 | 0.089 |
| SCR | CS+ | log | not ranked | AVE ACQ | 1st trial RI-Test | 0.659 | 0.123 | 0.418 | 0.900 | 5.361 | 105 | 0.000 | 0.216 | 0.275 |
| SCR | CS+ | log | not ranked | AVE last 2 trials ACQ | 1st trial RI-Test | 0.418 | 0.135 | 0.153 | 0.683 | 3.106 | 105 | 0.002 | 0.112 | 0.126 |
| SCR | CS+ | log | not ranked | 1st trial EXT | 1st trial RI-Test | 0.603 | 0.096 | 0.415 | 0.791 | 6.255 | 105 | 0.000 | 0.280 | 0.390 |
| SCR | CS+ | log | not ranked | AVE EXT | 1st trial RI-Test | 1.032 | 0.219 | 0.603 | 1.461 | 4.706 | 105 | 0.000 | 0.165 | 0.198 |
| SCR | CS+ | log | not ranked | AVE last 2 trials EXT | 1st trial RI-Test | 0.364 | 0.214 | -0.055 | 0.783 | 1.701 | 105 | 0.092 | 0.023 | 0.024 |
| SCR | CS- | log | not ranked | AVE ACQ | 1st trial EXT | 0.712 | 0.183 | 0.353 | 1.071 | 3.904 | 105 | 0.000 | 0.133 | 0.154 |
| SCR | CS- | log | not ranked | AVE last 2 trials ACQ | 1st trial EXT | 0.518 | 0.146 | 0.232 | 0.804 | 3.546 | 105 | 0.001 | 0.089 | 0.098 |
| SCR | CS- | log | not ranked | AVE ACQ | AVE EXT | 0.384 | 0.081 | 0.225 | 0.543 | 4.729 | 105 | 0.000 | 0.295 | 0.418 |
| SCR | CS- | log | not ranked | AVE last 2 trials ACQ | AVE EXT | 0.245 | 0.073 | 0.102 | 0.388 | 3.356 | 105 | 0.001 | 0.152 | 0.179 |
| SCR | CS- | log | not ranked | AVE ACQ | AVE last 2 trials EXT | 0.379 | 0.145 | 0.095 | 0.663 | 2.608 | 105 | 0.010 | 0.175 | 0.213 |
| SCR | CS- | log | not ranked | AVE last 2 trials ACQ | AVE last 2 trials EXT | 0.285 | 0.106 | 0.077 | 0.493 | 2.687 | 105 | 0.008 | 0.126 | 0.144 |
| SCR | CS- | log | not ranked | AVE ACQ | 1st trial RI-Test | 0.612 | 0.216 | 0.189 | 1.035 | 2.833 | 105 | 0.006 | 0.086 | 0.094 |
| SCR | CS- | log | not ranked | AVE last 2 trials ACQ | 1st trial RI-Test | 0.449 | 0.213 | 0.032 | 0.866 | 2.108 | 105 | 0.037 | 0.058 | 0.062 |
| SCR | CS- | log | not ranked | 1st trial EXT | 1st trial RI-Test | 0.341 | 0.113 | 0.120 | 0.562 | 3.021 | 105 | 0.003 | 0.101 | 0.113 |
| SCR | CS- | log | not ranked | AVE EXT | 1st trial RI-Test | 0.578 | 0.280 | 0.029 | 1.127 | 2.066 | 105 | 0.041 | 0.038 | 0.040 |
| SCR | CS- | log | not ranked | AVE last 2 trials EXT | 1st trial RI-Test | 0.163 | 0.287 | -0.400 | 0.726 | 0.568 | 105 | 0.571 | 0.005 | 0.005 |
| SCR | CS dis. | log rc | not ranked | AVE ACQ | 1st trial EXT | 0.378 | 0.200 | -0.014 | 0.770 | 1.894 | 105 | 0.061 | 0.030 | 0.031 |
| SCR | CS dis. | log rc | not ranked | AVE last 2 trials ACQ | 1st trial EXT | 0.298 | 0.109 | 0.084 | 0.512 | 2.733 | 105 | 0.007 | 0.046 | 0.049 |
| SCR | CS dis. | log rc | not ranked | AVE ACQ | AVE EXT | 0.071 | 0.054 | -0.035 | 0.177 | 1.328 | 105 | 0.187 | 0.017 | 0.017 |
| SCR | CS dis. | log rc | not ranked | AVE last 2 trials ACQ | AVE EXT | 0.022 | 0.038 | -0.052 | 0.096 | 0.572 | 105 | 0.568 | 0.004 | 0.004 |
| SCR | CS dis. | log rc | not ranked | AVE ACQ | AVE last 2 trials EXT | 0.103 | 0.106 | -0.105 | 0.311 | 0.974 | 105 | 0.332 | 0.010 | 0.010 |
| SCR | CS dis. | log rc | not ranked | AVE last 2 trials ACQ | AVE last 2 trials EXT | -0.007 | 0.093 | -0.189 | 0.175 | -0.073 | 105 | 0.942 | 0.000 | 0.000 |
| SCR | CS dis. | log rc | not ranked | AVE ACQ | 1st trial RI-Test | -0.212 | 0.225 | -0.653 | 0.229 | -0.943 | 105 | 0.348 | 0.009 | 0.009 |
| SCR | CS dis. | log rc | not ranked | AVE last 2 trials ACQ | 1st trial RI-Test | -0.041 | 0.158 | -0.351 | 0.269 | -0.259 | 105 | 0.796 | 0.001 | 0.001 |
| SCR | CS dis. | log rc | not ranked | 1st trial EXT | 1st trial RI-Test | 0.032 | 0.112 | -0.188 | 0.252 | 0.290 | 105 | 0.773 | 0.001 | 0.001 |
| SCR | CS dis. | log rc | not ranked | AVE EXT | 1st trial RI-Test | -0.025 | 0.381 | -0.772 | 0.722 | -0.066 | 105 | 0.948 | 0.000 | 0.000 |
| SCR | CS dis. | log rc | not ranked | AVE last 2 trials EXT | 1st trial RI-Test | -0.360 | 0.376 | -1.097 | 0.377 | -0.958 | 105 | 0.340 | 0.027 | 0.028 |
| SCR | CS+ | log rc | not ranked | AVE ACQ | 1st trial EXT | 0.435 | 0.122 | 0.196 | 0.674 | 3.564 | 105 | 0.001 | 0.108 | 0.121 |
| SCR | CS+ | log rc | not ranked | AVE last 2 trials ACQ | 1st trial EXT | 0.320 | 0.111 | 0.102 | 0.538 | 2.886 | 105 | 0.005 | 0.069 | 0.074 |
| SCR | CS+ | log rc | not ranked | AVE ACQ | AVE EXT | 0.267 | 0.058 | 0.153 | 0.381 | 4.578 | 105 | 0.000 | 0.215 | 0.274 |
| SCR | CS+ | log rc | not ranked | AVE last 2 trials ACQ | AVE EXT | 0.239 | 0.059 | 0.123 | 0.355 | 4.069 | 105 | 0.000 | 0.204 | 0.256 |
| SCR | CS+ | log rc | not ranked | AVE ACQ | AVE last 2 trials EXT | 0.181 | 0.073 | 0.038 | 0.324 | 2.495 | 105 | 0.014 | 0.079 | 0.086 |
| SCR | CS+ | log rc | not ranked | AVE last 2 trials ACQ | AVE last 2 trials EXT | 0.157 | 0.069 | 0.022 | 0.292 | 2.268 | 105 | 0.025 | 0.070 | 0.075 |
| SCR | CS+ | log rc | not ranked | AVE ACQ | 1st trial RI-Test | 0.233 | 0.138 | -0.037 | 0.503 | 1.681 | 105 | 0.096 | 0.023 | 0.024 |
| SCR | CS+ | log rc | not ranked | AVE last 2 trials ACQ | 1st trial RI-Test | 0.070 | 0.130 | -0.185 | 0.325 | 0.543 | 105 | 0.588 | 0.003 | 0.003 |
| SCR | CS+ | log rc | not ranked | 1st trial EXT | 1st trial RI-Test | 0.403 | 0.103 | 0.201 | 0.605 | 3.910 | 105 | 0.000 | 0.122 | 0.138 |
| SCR | CS+ | log rc | not ranked | AVE EXT | 1st trial RI-Test | 0.500 | 0.206 | 0.096 | 0.904 | 2.425 | 105 | 0.017 | 0.035 | 0.037 |
| SCR | CS+ | log rc | not ranked | AVE last 2 trials EXT | 1st trial RI-Test | -0.021 | 0.196 | -0.405 | 0.363 | -0.106 | 105 | 0.916 | 0.000 | 0.000 |
| SCR | CS- | log rc | not ranked | AVE ACQ | 1st trial EXT | 0.247 | 0.193 | -0.131 | 0.625 | 1.278 | 105 | 0.204 | 0.014 | 0.014 |
| SCR | CS- | log rc | not ranked | AVE last 2 trials ACQ | 1st trial EXT | 0.192 | 0.150 | -0.102 | 0.486 | 1.275 | 105 | 0.205 | 0.010 | 0.011 |
| SCR | CS- | log rc | not ranked | AVE ACQ | AVE EXT | 0.370 | 0.078 | 0.217 | 0.523 | 4.719 | 105 | 0.000 | 0.272 | 0.375 |
| SCR | CS- | log rc | not ranked | AVE last 2 trials ACQ | AVE EXT | 0.246 | 0.069 | 0.111 | 0.381 | 3.577 | 105 | 0.001 | 0.151 | 0.178 |
| SCR | CS- | log rc | not ranked | AVE ACQ | AVE last 2 trials EXT | 0.310 | 0.104 | 0.106 | 0.514 | 2.971 | 105 | 0.004 | 0.125 | 0.143 |
| SCR | CS- | log rc | not ranked | AVE last 2 trials ACQ | AVE last 2 trials EXT | 0.246 | 0.078 | 0.093 | 0.399 | 3.143 | 105 | 0.002 | 0.099 | 0.110 |
| SCR | CS- | log rc | not ranked | AVE ACQ | 1st trial RI-Test | 0.397 | 0.240 | -0.073 | 0.867 | 1.654 | 105 | 0.101 | 0.031 | 0.032 |
| SCR | CS- | log rc | not ranked | AVE last 2 trials ACQ | 1st trial RI-Test | 0.255 | 0.216 | -0.168 | 0.678 | 1.179 | 105 | 0.241 | 0.016 | 0.017 |
| SCR | CS- | log rc | not ranked | 1st trial EXT | 1st trial RI-Test | 0.192 | 0.118 | -0.039 | 0.423 | 1.619 | 105 | 0.108 | 0.032 | 0.033 |
| SCR | CS- | log rc | not ranked | AVE EXT | 1st trial RI-Test | 0.178 | 0.278 | -0.367 | 0.723 | 0.639 | 105 | 0.524 | 0.003 | 0.003 |
| SCR | CS- | log rc | not ranked | AVE last 2 trials EXT | 1st trial RI-Test | -0.108 | 0.189 | -0.478 | 0.262 | -0.569 | 105 | 0.571 | 0.002 | 0.002 |
| SCR | CS dis. | raw | ranked | AVE ACQ | 1st trial EXT | 0.180 | 0.089 | 0.006 | 0.354 | 2.009 | 105 | 0.047 | 0.032 | 0.033 |
| SCR | CS dis. | raw | ranked | AVE last 2 trials ACQ | 1st trial EXT | 0.273 | 0.086 | 0.104 | 0.442 | 3.167 | 105 | 0.002 | 0.087 | 0.095 |
| SCR | CS dis. | raw | ranked | AVE ACQ | AVE EXT | 0.211 | 0.097 | 0.021 | 0.401 | 2.169 | 105 | 0.032 | 0.043 | 0.045 |
| SCR | CS dis. | raw | ranked | AVE last 2 trials ACQ | AVE EXT | 0.228 | 0.092 | 0.048 | 0.408 | 2.489 | 105 | 0.014 | 0.059 | 0.063 |
| SCR | CS dis. | raw | ranked | AVE ACQ | AVE last 2 trials EXT | 0.125 | 0.120 | -0.110 | 0.360 | 1.045 | 105 | 0.299 | 0.012 | 0.012 |
| SCR | CS dis. | raw | ranked | AVE last 2 trials ACQ | AVE last 2 trials EXT | 0.200 | 0.106 | -0.008 | 0.408 | 1.888 | 105 | 0.062 | 0.036 | 0.038 |
| SCR | CS dis. | raw | ranked | AVE ACQ | 1st trial RI-Test | 0.037 | 0.103 | -0.165 | 0.239 | 0.362 | 105 | 0.718 | 0.001 | 0.001 |
| SCR | CS dis. | raw | ranked | AVE last 2 trials ACQ | 1st trial RI-Test | -0.071 | 0.096 | -0.259 | 0.117 | -0.740 | 105 | 0.461 | 0.006 | 0.006 |
| SCR | CS dis. | raw | ranked | 1st trial EXT | 1st trial RI-Test | 0.034 | 0.112 | -0.186 | 0.254 | 0.303 | 105 | 0.763 | 0.001 | 0.001 |
| SCR | CS dis. | raw | ranked | AVE EXT | 1st trial RI-Test | 0.017 | 0.109 | -0.197 | 0.231 | 0.154 | 105 | 0.878 | 0.000 | 0.000 |
| SCR | CS dis. | raw | ranked | AVE last 2 trials EXT | 1st trial RI-Test | 0.068 | 0.087 | -0.103 | 0.239 | 0.773 | 105 | 0.442 | 0.006 | 0.006 |
| SCR | CS+ | raw | ranked | AVE ACQ | 1st trial EXT | 0.594 | 0.075 | 0.447 | 0.741 | 7.958 | 105 | 0.000 | 0.319 | 0.469 |
| SCR | CS+ | raw | ranked | AVE last 2 trials ACQ | 1st trial EXT | 0.381 | 0.078 | 0.228 | 0.534 | 4.860 | 105 | 0.000 | 0.187 | 0.230 |
| SCR | CS+ | raw | ranked | AVE ACQ | AVE EXT | 0.607 | 0.071 | 0.468 | 0.746 | 8.500 | 105 | 0.000 | 0.324 | 0.480 |
| SCR | CS+ | raw | ranked | AVE last 2 trials ACQ | AVE EXT | 0.451 | 0.077 | 0.300 | 0.602 | 5.852 | 105 | 0.000 | 0.256 | 0.343 |
| SCR | CS+ | raw | ranked | AVE ACQ | AVE last 2 trials EXT | 0.364 | 0.125 | 0.119 | 0.609 | 2.912 | 105 | 0.004 | 0.072 | 0.078 |
| SCR | CS+ | raw | ranked | AVE last 2 trials ACQ | AVE last 2 trials EXT | 0.281 | 0.108 | 0.069 | 0.493 | 2.608 | 105 | 0.010 | 0.061 | 0.065 |
| SCR | CS+ | raw | ranked | AVE ACQ | 1st trial RI-Test | 0.485 | 0.083 | 0.322 | 0.648 | 5.828 | 105 | 0.000 | 0.215 | 0.274 |
| SCR | CS+ | raw | ranked | AVE last 2 trials ACQ | 1st trial RI-Test | 0.216 | 0.088 | 0.044 | 0.388 | 2.441 | 105 | 0.016 | 0.061 | 0.064 |
| SCR | CS+ | raw | ranked | 1st trial EXT | 1st trial RI-Test | 0.518 | 0.083 | 0.355 | 0.681 | 6.282 | 105 | 0.000 | 0.272 | 0.374 |
| SCR | CS+ | raw | ranked | AVE EXT | 1st trial RI-Test | 0.340 | 0.097 | 0.150 | 0.530 | 3.507 | 105 | 0.001 | 0.120 | 0.136 |
| SCR | CS+ | raw | ranked | AVE last 2 trials EXT | 1st trial RI-Test | 0.009 | 0.075 | -0.138 | 0.156 | 0.113 | 105 | 0.910 | 0.000 | 0.000 |
| SCR | CS- | raw | ranked | AVE ACQ | 1st trial EXT | 0.388 | 0.096 | 0.200 | 0.576 | 4.057 | 105 | 0.000 | 0.129 | 0.148 |
| SCR | CS- | raw | ranked | AVE last 2 trials ACQ | 1st trial EXT | 0.196 | 0.078 | 0.043 | 0.349 | 2.507 | 105 | 0.014 | 0.056 | 0.060 |
| SCR | CS- | raw | ranked | AVE ACQ | AVE EXT | 0.670 | 0.070 | 0.533 | 0.807 | 9.586 | 105 | 0.000 | 0.384 | 0.623 |
| SCR | CS- | raw | ranked | AVE last 2 trials ACQ | AVE EXT | 0.353 | 0.072 | 0.212 | 0.494 | 4.905 | 105 | 0.000 | 0.184 | 0.225 |
| SCR | CS- | raw | ranked | AVE ACQ | AVE last 2 trials EXT | 0.427 | 0.115 | 0.202 | 0.652 | 3.702 | 105 | 0.000 | 0.109 | 0.122 |
| SCR | CS- | raw | ranked | AVE last 2 trials ACQ | AVE last 2 trials EXT | 0.388 | 0.094 | 0.204 | 0.572 | 4.117 | 105 | 0.000 | 0.155 | 0.183 |
| SCR | CS- | raw | ranked | AVE ACQ | 1st trial RI-Test | 0.340 | 0.104 | 0.136 | 0.544 | 3.281 | 105 | 0.001 | 0.099 | 0.110 |
| SCR | CS- | raw | ranked | AVE last 2 trials ACQ | 1st trial RI-Test | 0.206 | 0.080 | 0.049 | 0.363 | 2.567 | 105 | 0.012 | 0.062 | 0.067 |
| SCR | CS- | raw | ranked | 1st trial EXT | 1st trial RI-Test | 0.327 | 0.100 | 0.131 | 0.523 | 3.265 | 105 | 0.001 | 0.107 | 0.119 |
| SCR | CS- | raw | ranked | AVE EXT | 1st trial RI-Test | 0.298 | 0.096 | 0.110 | 0.486 | 3.096 | 105 | 0.003 | 0.089 | 0.097 |
| SCR | CS- | raw | ranked | AVE last 2 trials EXT | 1st trial RI-Test | 0.110 | 0.069 | -0.025 | 0.245 | 1.583 | 105 | 0.117 | 0.017 | 0.018 |
| SCR | CS dis. | log | ranked | AVE ACQ | 1st trial EXT | 0.177 | 0.090 | 0.001 | 0.353 | 1.971 | 105 | 0.051 | 0.031 | 0.032 |
| SCR | CS dis. | log | ranked | AVE last 2 trials ACQ | 1st trial EXT | 0.269 | 0.086 | 0.100 | 0.438 | 3.135 | 105 | 0.002 | 0.084 | 0.092 |
| SCR | CS dis. | log | ranked | AVE ACQ | AVE EXT | 0.206 | 0.098 | 0.014 | 0.398 | 2.108 | 105 | 0.037 | 0.041 | 0.043 |
| SCR | CS dis. | log | ranked | AVE last 2 trials ACQ | AVE EXT | 0.214 | 0.092 | 0.034 | 0.394 | 2.319 | 105 | 0.022 | 0.052 | 0.055 |
| SCR | CS dis. | log | ranked | AVE ACQ | AVE last 2 trials EXT | 0.132 | 0.120 | -0.103 | 0.367 | 1.102 | 105 | 0.273 | 0.014 | 0.014 |
| SCR | CS dis. | log | ranked | AVE last 2 trials ACQ | AVE last 2 trials EXT | 0.194 | 0.106 | -0.014 | 0.402 | 1.838 | 105 | 0.069 | 0.034 | 0.036 |
| SCR | CS dis. | log | ranked | AVE ACQ | 1st trial RI-Test | 0.039 | 0.103 | -0.163 | 0.241 | 0.380 | 105 | 0.704 | 0.002 | 0.002 |
| SCR | CS dis. | log | ranked | AVE last 2 trials ACQ | 1st trial RI-Test | -0.081 | 0.096 | -0.269 | 0.107 | -0.845 | 105 | 0.400 | 0.008 | 0.008 |
| SCR | CS dis. | log | ranked | 1st trial EXT | 1st trial RI-Test | 0.030 | 0.110 | -0.186 | 0.246 | 0.270 | 105 | 0.787 | 0.001 | 0.001 |
| SCR | CS dis. | log | ranked | AVE EXT | 1st trial RI-Test | 0.009 | 0.109 | -0.205 | 0.223 | 0.084 | 105 | 0.933 | 0.000 | 0.000 |
| SCR | CS dis. | log | ranked | AVE last 2 trials EXT | 1st trial RI-Test | 0.060 | 0.087 | -0.111 | 0.231 | 0.696 | 105 | 0.488 | 0.005 | 0.005 |
| SCR | CS+ | log | ranked | AVE ACQ | 1st trial EXT | 0.591 | 0.075 | 0.444 | 0.738 | 7.906 | 105 | 0.000 | 0.316 | 0.462 |
| SCR | CS+ | log | ranked | AVE last 2 trials ACQ | 1st trial EXT | 0.382 | 0.078 | 0.229 | 0.535 | 4.880 | 105 | 0.000 | 0.188 | 0.231 |
| SCR | CS+ | log | ranked | AVE ACQ | AVE EXT | 0.606 | 0.073 | 0.463 | 0.749 | 8.363 | 105 | 0.000 | 0.324 | 0.479 |
| SCR | CS+ | log | ranked | AVE last 2 trials ACQ | AVE EXT | 0.455 | 0.077 | 0.304 | 0.606 | 5.942 | 105 | 0.000 | 0.260 | 0.351 |
| SCR | CS+ | log | ranked | AVE ACQ | AVE last 2 trials EXT | 0.375 | 0.125 | 0.130 | 0.620 | 3.003 | 105 | 0.003 | 0.077 | 0.083 |
| SCR | CS+ | log | ranked | AVE last 2 trials ACQ | AVE last 2 trials EXT | 0.285 | 0.108 | 0.073 | 0.497 | 2.646 | 105 | 0.009 | 0.063 | 0.067 |
| SCR | CS+ | log | ranked | AVE ACQ | 1st trial RI-Test | 0.484 | 0.084 | 0.319 | 0.649 | 5.789 | 105 | 0.000 | 0.214 | 0.272 |
| SCR | CS+ | log | ranked | AVE last 2 trials ACQ | 1st trial RI-Test | 0.221 | 0.088 | 0.049 | 0.393 | 2.514 | 105 | 0.013 | 0.063 | 0.068 |
| SCR | CS+ | log | ranked | 1st trial EXT | 1st trial RI-Test | 0.518 | 0.083 | 0.355 | 0.681 | 6.282 | 105 | 0.000 | 0.272 | 0.374 |
| SCR | CS+ | log | ranked | AVE EXT | 1st trial RI-Test | 0.337 | 0.097 | 0.147 | 0.527 | 3.488 | 105 | 0.001 | 0.118 | 0.134 |
| SCR | CS+ | log | ranked | AVE last 2 trials EXT | 1st trial RI-Test | 0.008 | 0.075 | -0.139 | 0.155 | 0.110 | 105 | 0.912 | 0.000 | 0.000 |
| SCR | CS- | log | ranked | AVE ACQ | 1st trial EXT | 0.387 | 0.095 | 0.201 | 0.573 | 4.057 | 105 | 0.000 | 0.128 | 0.147 |
| SCR | CS- | log | ranked | AVE last 2 trials ACQ | 1st trial EXT | 0.197 | 0.078 | 0.044 | 0.350 | 2.520 | 105 | 0.013 | 0.057 | 0.060 |
| SCR | CS- | log | ranked | AVE ACQ | AVE EXT | 0.674 | 0.070 | 0.537 | 0.811 | 9.688 | 105 | 0.000 | 0.388 | 0.634 |
| SCR | CS- | log | ranked | AVE last 2 trials ACQ | AVE EXT | 0.356 | 0.072 | 0.215 | 0.497 | 4.959 | 105 | 0.000 | 0.187 | 0.230 |
| SCR | CS- | log | ranked | AVE ACQ | AVE last 2 trials EXT | 0.432 | 0.115 | 0.207 | 0.657 | 3.751 | 105 | 0.000 | 0.111 | 0.125 |
| SCR | CS- | log | ranked | AVE last 2 trials ACQ | AVE last 2 trials EXT | 0.391 | 0.094 | 0.207 | 0.575 | 4.151 | 105 | 0.000 | 0.157 | 0.186 |
| SCR | CS- | log | ranked | AVE ACQ | 1st trial RI-Test | 0.341 | 0.104 | 0.137 | 0.545 | 3.289 | 105 | 0.001 | 0.099 | 0.110 |
| SCR | CS- | log | ranked | AVE last 2 trials ACQ | 1st trial RI-Test | 0.206 | 0.080 | 0.049 | 0.363 | 2.573 | 105 | 0.011 | 0.063 | 0.067 |
| SCR | CS- | log | ranked | 1st trial EXT | 1st trial RI-Test | 0.327 | 0.100 | 0.131 | 0.523 | 3.265 | 105 | 0.001 | 0.107 | 0.119 |
| SCR | CS- | log | ranked | AVE EXT | 1st trial RI-Test | 0.298 | 0.096 | 0.110 | 0.486 | 3.108 | 105 | 0.002 | 0.089 | 0.097 |
| SCR | CS- | log | ranked | AVE last 2 trials EXT | 1st trial RI-Test | 0.109 | 0.069 | -0.026 | 0.244 | 1.578 | 105 | 0.118 | 0.017 | 0.017 |
| SCR | CS dis. | log rc | ranked | AVE ACQ | 1st trial EXT | 0.150 | 0.096 | -0.038 | 0.338 | 1.571 | 105 | 0.119 | 0.023 | 0.023 |
| SCR | CS dis. | log rc | ranked | AVE last 2 trials ACQ | 1st trial EXT | 0.248 | 0.085 | 0.081 | 0.415 | 2.930 | 105 | 0.004 | 0.071 | 0.077 |
| SCR | CS dis. | log rc | ranked | AVE ACQ | AVE EXT | 0.136 | 0.096 | -0.052 | 0.324 | 1.411 | 105 | 0.161 | 0.018 | 0.018 |
| SCR | CS dis. | log rc | ranked | AVE last 2 trials ACQ | AVE EXT | 0.164 | 0.094 | -0.020 | 0.348 | 1.739 | 105 | 0.085 | 0.031 | 0.032 |
| SCR | CS dis. | log rc | ranked | AVE ACQ | AVE last 2 trials EXT | 0.135 | 0.120 | -0.100 | 0.370 | 1.130 | 105 | 0.261 | 0.014 | 0.015 |
| SCR | CS dis. | log rc | ranked | AVE last 2 trials ACQ | AVE last 2 trials EXT | 0.167 | 0.105 | -0.039 | 0.373 | 1.594 | 105 | 0.114 | 0.025 | 0.026 |
| SCR | CS dis. | log rc | ranked | AVE ACQ | 1st trial RI-Test | -0.038 | 0.100 | -0.234 | 0.158 | -0.381 | 105 | 0.704 | 0.001 | 0.001 |
| SCR | CS dis. | log rc | ranked | AVE last 2 trials ACQ | 1st trial RI-Test | -0.099 | 0.093 | -0.281 | 0.083 | -1.064 | 105 | 0.290 | 0.011 | 0.012 |
| SCR | CS dis. | log rc | ranked | 1st trial EXT | 1st trial RI-Test | 0.040 | 0.100 | -0.156 | 0.236 | 0.399 | 105 | 0.691 | 0.002 | 0.002 |
| SCR | CS dis. | log rc | ranked | AVE EXT | 1st trial RI-Test | -0.014 | 0.101 | -0.212 | 0.184 | -0.137 | 105 | 0.892 | 0.000 | 0.000 |
| SCR | CS dis. | log rc | ranked | AVE last 2 trials EXT | 1st trial RI-Test | -0.010 | 0.084 | -0.175 | 0.155 | -0.121 | 105 | 0.904 | 0.000 | 0.000 |
| SCR | CS+ | log rc | ranked | AVE ACQ | 1st trial EXT | 0.358 | 0.096 | 0.170 | 0.546 | 3.722 | 105 | 0.000 | 0.116 | 0.131 |
| SCR | CS+ | log rc | ranked | AVE last 2 trials ACQ | 1st trial EXT | 0.244 | 0.082 | 0.083 | 0.405 | 2.957 | 105 | 0.004 | 0.076 | 0.083 |
| SCR | CS+ | log rc | ranked | AVE ACQ | AVE EXT | 0.558 | 0.089 | 0.384 | 0.732 | 6.264 | 105 | 0.000 | 0.274 | 0.377 |
| SCR | CS+ | log rc | ranked | AVE last 2 trials ACQ | AVE EXT | 0.437 | 0.076 | 0.288 | 0.586 | 5.786 | 105 | 0.000 | 0.240 | 0.316 |
| SCR | CS+ | log rc | ranked | AVE ACQ | AVE last 2 trials EXT | 0.397 | 0.131 | 0.140 | 0.654 | 3.044 | 105 | 0.003 | 0.086 | 0.094 |
| SCR | CS+ | log rc | ranked | AVE last 2 trials ACQ | AVE last 2 trials EXT | 0.299 | 0.110 | 0.083 | 0.515 | 2.729 | 105 | 0.007 | 0.069 | 0.075 |
| SCR | CS+ | log rc | ranked | AVE ACQ | 1st trial RI-Test | 0.200 | 0.097 | 0.010 | 0.390 | 2.058 | 105 | 0.042 | 0.037 | 0.038 |
| SCR | CS+ | log rc | ranked | AVE last 2 trials ACQ | 1st trial RI-Test | 0.074 | 0.085 | -0.093 | 0.241 | 0.869 | 105 | 0.387 | 0.007 | 0.007 |
| SCR | CS+ | log rc | ranked | 1st trial EXT | 1st trial RI-Test | 0.349 | 0.097 | 0.159 | 0.539 | 3.587 | 105 | 0.001 | 0.124 | 0.141 |
| SCR | CS+ | log rc | ranked | AVE EXT | 1st trial RI-Test | 0.161 | 0.096 | -0.027 | 0.349 | 1.681 | 105 | 0.096 | 0.027 | 0.028 |
| SCR | CS+ | log rc | ranked | AVE last 2 trials EXT | 1st trial RI-Test | -0.067 | 0.071 | -0.206 | 0.072 | -0.937 | 105 | 0.351 | 0.007 | 0.008 |
| SCR | CS- | log rc | ranked | AVE ACQ | 1st trial EXT | 0.244 | 0.100 | 0.048 | 0.440 | 2.446 | 105 | 0.016 | 0.051 | 0.053 |
| SCR | CS- | log rc | ranked | AVE last 2 trials ACQ | 1st trial EXT | 0.111 | 0.078 | -0.042 | 0.264 | 1.418 | 105 | 0.159 | 0.018 | 0.018 |
| SCR | CS- | log rc | ranked | AVE ACQ | AVE EXT | 0.682 | 0.072 | 0.541 | 0.823 | 9.479 | 105 | 0.000 | 0.397 | 0.659 |
| SCR | CS- | log rc | ranked | AVE last 2 trials ACQ | AVE EXT | 0.347 | 0.071 | 0.208 | 0.486 | 4.913 | 105 | 0.000 | 0.177 | 0.215 |
| SCR | CS- | log rc | ranked | AVE ACQ | AVE last 2 trials EXT | 0.487 | 0.117 | 0.258 | 0.716 | 4.148 | 105 | 0.000 | 0.141 | 0.164 |
| SCR | CS- | log rc | ranked | AVE last 2 trials ACQ | AVE last 2 trials EXT | 0.383 | 0.093 | 0.201 | 0.565 | 4.107 | 105 | 0.000 | 0.150 | 0.177 |
| SCR | CS- | log rc | ranked | AVE ACQ | 1st trial RI-Test | 0.251 | 0.097 | 0.061 | 0.441 | 2.582 | 105 | 0.011 | 0.054 | 0.057 |
| SCR | CS- | log rc | ranked | AVE last 2 trials ACQ | 1st trial RI-Test | 0.146 | 0.080 | -0.011 | 0.303 | 1.815 | 105 | 0.072 | 0.031 | 0.032 |
| SCR | CS- | log rc | ranked | 1st trial EXT | 1st trial RI-Test | 0.189 | 0.104 | -0.015 | 0.393 | 1.822 | 105 | 0.071 | 0.036 | 0.037 |
| SCR | CS- | log rc | ranked | AVE EXT | 1st trial RI-Test | 0.145 | 0.100 | -0.051 | 0.341 | 1.454 | 105 | 0.149 | 0.021 | 0.022 |
| SCR | CS- | log rc | ranked | AVE last 2 trials EXT | 1st trial RI-Test | 0.039 | 0.070 | -0.098 | 0.176 | 0.556 | 105 | 0.579 | 0.002 | 0.002 |
| *Note*. Ampl. = Amplitude, Stim. = Stimulus, CI = Confidence Interval, CS dis. = CS discrimination, log = log-transformed, log rc = log-transformed and range corrected, AVE = average, ACQ = Acquisition training, EXT = Extinction training, RI = Reinstatement, RI-Test = Reinstatement-Test. | | | | | | | | | | | | | | |

**References**

Cohen, J. (1988). *Statistical power analysis for the behavioral sciences* (2nd ed). L. Erlbaum Associates.

Selya, A. S., Rose, J. S., Dierker, L. C., Hedeker, D., & Mermelstein, R. J. (2012). A Practical Guide to Calculating Cohen’s f2, a Measure of Local Effect Size, from PROC MIXED. *Frontiers in Psychology*, *3*. <https://doi.org/10.3389/fpsyg.2012.00111>
