# Supplementary material for: Robust group- but limited individual-level (longitudinal) reliability and insights into cross-phases response prediction of conditioned fear
Source: eLife. 2022 Sep 13;11:e78717. doi: 10.7554/eLife.78717 (PMC9691022; doi:10.7554/eLife.78717)
Supplement: Supplementary file 8. [file elife-78717-supp8.docx]

# Detailed results of predictability analysis: fear ratings

Cohen’s f^2^ (formula: f^2^ = R^2^/1 – R^2^) was calculated as effect size. According to the guidelines of Cohen (1988), f^2^ $\geq$ .02, f^2^ $\geq$ .15 and f^2^ $\geq$ .34 represent small, medium and large effect sizes respectively. Since Cohen’s f^2^ is informative, but less common (Selya, Rose, Dierker, Hedeker, & Mermelstein, 2012), additionally R squared is reported as effect size.

**Supplementary File 8:** Detailed results of linear regressions: fear ratings.

| **Outcome** | **Stim.-type** | **Ranking** | **Predictor** | **Criterion** | ***b*** | ***SE_b_*** | **Lower 95% CI** | **Upper 95% CI** | ***t*** | ***df*** | ***p*** | ***R^2^*** | ***Cohen's f^2^*** |
| --- | --- | --- | --- | --- | --- | --- | --- | --- | --- | --- | --- | --- | --- |
| Fear Ratings | CS dis. | not ranked | post-pre ACQ | pre EXT | 0.519 | 0.094 | 0.335 | 0.703 | 5.543 | 77 | 0.000 | 0.270 | 0.369 |
| Fear Ratings | CS dis. | not ranked | post ACQ | pre EXT | 0.570 | 0.088 | 0.398 | 0.742 | 6.454 | 92 | 0.000 | 0.265 | 0.360 |
| Fear Ratings | CS dis. | not ranked | post-pre ACQ | pre-post EXT | 0.372 | 0.132 | 0.113 | 0.631 | 2.827 | 76 | 0.006 | 0.175 | 0.213 |
| Fear Ratings | CS dis. | not ranked | post ACQ | pre-post EXT | 0.413 | 0.107 | 0.203 | 0.623 | 3.851 | 91 | 0.000 | 0.177 | 0.215 |
| Fear Ratings | CS dis. | not ranked | post-pre ACQ | post EXT | 0.144 | 0.087 | -0.027 | 0.315 | 1.648 | 79 | 0.103 | 0.076 | 0.082 |
| Fear Ratings | CS dis. | not ranked | post ACQ | post EXT | 0.139 | 0.077 | -0.012 | 0.290 | 1.818 | 98 | 0.072 | 0.063 | 0.068 |
| Fear Ratings | CS dis. | not ranked | post ACQ | 1st trial RI-Test | 0.257 | 0.084 | 0.092 | 0.422 | 3.056 | 74 | 0.003 | 0.099 | 0.110 |
| Fear Ratings | CS dis. | not ranked | post-pre ACQ | 1st trial RI-Test | 0.240 | 0.081 | 0.081 | 0.399 | 2.967 | 60 | 0.004 | 0.117 | 0.132 |
| Fear Ratings | CS dis. | not ranked | pre EXT | 1st trial RI-Test | 0.236 | 0.086 | 0.067 | 0.405 | 2.739 | 69 | 0.008 | 0.112 | 0.127 |
| Fear Ratings | CS dis. | not ranked | pre-post EXT | 1st trial RI-Test | 0.187 | 0.104 | -0.017 | 0.391 | 1.793 | 68 | 0.077 | 0.052 | 0.055 |
| Fear Ratings | CS dis. | not ranked | post EXT | 1st trial RI-Test | 0.301 | 0.187 | -0.066 | 0.668 | 1.610 | 71 | 0.112 | 0.043 | 0.045 |
| Fear Ratings | CS+ | not ranked | post-pre ACQ | pre EXT | 0.509 | 0.095 | 0.323 | 0.695 | 5.365 | 91 | 0.000 | 0.208 | 0.263 |
| Fear Ratings | CS+ | not ranked | post ACQ | pre EXT | 0.655 | 0.090 | 0.479 | 0.831 | 7.307 | 97 | 0.000 | 0.319 | 0.469 |
| Fear Ratings | CS+ | not ranked | post-pre ACQ | pre-post EXT | 0.425 | 0.081 | 0.266 | 0.584 | 5.218 | 90 | 0.000 | 0.194 | 0.241 |
| Fear Ratings | CS+ | not ranked | post ACQ | pre-post EXT | 0.461 | 0.083 | 0.298 | 0.624 | 5.547 | 96 | 0.000 | 0.209 | 0.263 |
| Fear Ratings | CS+ | not ranked | post-pre ACQ | post EXT | 0.084 | 0.069 | -0.051 | 0.219 | 1.212 | 92 | 0.229 | 0.011 | 0.011 |
| Fear Ratings | CS+ | not ranked | post ACQ | post EXT | 0.172 | 0.073 | 0.029 | 0.315 | 2.370 | 101 | 0.020 | 0.042 | 0.044 |
| Fear Ratings | CS+ | not ranked | post ACQ | 1st trial RI-Test | 0.503 | 0.102 | 0.303 | 0.703 | 4.928 | 85 | 0.000 | 0.171 | 0.207 |
| Fear Ratings | CS+ | not ranked | post-pre ACQ | 1st trial RI-Test | 0.330 | 0.105 | 0.124 | 0.536 | 3.153 | 79 | 0.002 | 0.091 | 0.100 |
| Fear Ratings | CS+ | not ranked | pre EXT | 1st trial RI-Test | 0.430 | 0.093 | 0.248 | 0.612 | 4.630 | 82 | 0.000 | 0.184 | 0.226 |
| Fear Ratings | CS+ | not ranked | pre-post EXT | 1st trial RI-Test | 0.293 | 0.119 | 0.060 | 0.526 | 2.455 | 81 | 0.016 | 0.060 | 0.064 |
| Fear Ratings | CS+ | not ranked | post EXT | 1st trial RI-Test | 0.352 | 0.134 | 0.089 | 0.615 | 2.615 | 84 | 0.011 | 0.072 | 0.077 |
| Fear Ratings | CS- | not ranked | post-pre ACQ | pre EXT | 0.077 | 0.099 | -0.117 | 0.271 | 0.773 | 86 | 0.442 | 0.019 | 0.020 |
| Fear Ratings | CS- | not ranked | post ACQ | pre EXT | 0.244 | 0.101 | 0.046 | 0.442 | 2.423 | 98 | 0.017 | 0.130 | 0.150 |
| Fear Ratings | CS- | not ranked | post-pre ACQ | pre-post EXT | -0.197 | 0.120 | -0.432 | 0.038 | -1.638 | 85 | 0.105 | 0.061 | 0.065 |
| Fear Ratings | CS- | not ranked | post ACQ | pre-post EXT | -0.147 | 0.123 | -0.388 | 0.094 | -1.198 | 97 | 0.234 | 0.030 | 0.031 |
| Fear Ratings | CS- | not ranked | post-pre ACQ | post EXT | 0.236 | 0.138 | -0.034 | 0.506 | 1.708 | 88 | 0.091 | 0.090 | 0.099 |
| Fear Ratings | CS- | not ranked | post ACQ | post EXT | 0.386 | 0.140 | 0.112 | 0.660 | 2.753 | 100 | 0.007 | 0.217 | 0.278 |
| Fear Ratings | CS- | not ranked | post ACQ | 1st trial RI-Test | 0.270 | 0.176 | -0.075 | 0.615 | 1.534 | 88 | 0.129 | 0.039 | 0.040 |
| Fear Ratings | CS- | not ranked | post-pre ACQ | 1st trial RI-Test | 0.082 | 0.160 | -0.232 | 0.396 | 0.513 | 78 | 0.610 | 0.004 | 0.004 |
| Fear Ratings | CS- | not ranked | pre EXT | 1st trial RI-Test | 0.493 | 0.275 | -0.046 | 1.032 | 1.788 | 86 | 0.077 | 0.043 | 0.045 |
| Fear Ratings | CS- | not ranked | pre-post EXT | 1st trial RI-Test | -0.279 | 0.213 | -0.696 | 0.138 | -1.308 | 85 | 0.194 | 0.028 | 0.029 |
| Fear Ratings | CS- | not ranked | post EXT | 1st trial RI-Test | 0.582 | 0.193 | 0.204 | 0.960 | 3.010 | 87 | 0.003 | 0.109 | 0.122 |
| Fear Ratings | CS dis. | ranked | post-pre ACQ | pre EXT | 0.664 | 0.112 | 0.444 | 0.884 | 5.936 | 77 | 0.000 | 0.299 | 0.427 |
| Fear Ratings | CS dis. | ranked | post ACQ | pre EXT | 0.534 | 0.081 | 0.375 | 0.693 | 6.569 | 92 | 0.000 | 0.300 | 0.428 |
| Fear Ratings | CS dis. | ranked | post-pre ACQ | pre-post EXT | 0.595 | 0.117 | 0.366 | 0.824 | 5.082 | 76 | 0.000 | 0.241 | 0.317 |
| Fear Ratings | CS dis. | ranked | post ACQ | pre-post EXT | 0.461 | 0.084 | 0.296 | 0.626 | 5.461 | 91 | 0.000 | 0.231 | 0.301 |
| Fear Ratings | CS dis. | ranked | post-pre ACQ | post EXT | 0.269 | 0.167 | -0.058 | 0.596 | 1.613 | 79 | 0.111 | 0.033 | 0.034 |
| Fear Ratings | CS dis. | ranked | post ACQ | post EXT | 0.241 | 0.120 | 0.006 | 0.476 | 2.003 | 98 | 0.048 | 0.040 | 0.042 |
| Fear Ratings | CS dis. | ranked | post ACQ | 1st trial RI-Test | 0.213 | 0.086 | 0.044 | 0.382 | 2.471 | 74 | 0.016 | 0.072 | 0.078 |
| Fear Ratings | CS dis. | ranked | post-pre ACQ | 1st trial RI-Test | 0.236 | 0.116 | 0.009 | 0.463 | 2.043 | 60 | 0.045 | 0.066 | 0.071 |
| Fear Ratings | CS dis. | ranked | pre EXT | 1st trial RI-Test | 0.217 | 0.094 | 0.033 | 0.401 | 2.309 | 69 | 0.024 | 0.075 | 0.082 |
| Fear Ratings | CS dis. | ranked | pre-post EXT | 1st trial RI-Test | 0.138 | 0.097 | -0.052 | 0.328 | 1.417 | 68 | 0.161 | 0.029 | 0.030 |
| Fear Ratings | CS dis. | ranked | post EXT | 1st trial RI-Test | 0.143 | 0.081 | -0.016 | 0.302 | 1.769 | 71 | 0.081 | 0.047 | 0.050 |
| Fear Ratings | CS+ | ranked | post-pre ACQ | pre EXT | 0.516 | 0.101 | 0.318 | 0.714 | 5.107 | 91 | 0.000 | 0.215 | 0.274 |
| Fear Ratings | CS+ | ranked | post ACQ | pre EXT | 0.584 | 0.087 | 0.413 | 0.755 | 6.675 | 97 | 0.000 | 0.326 | 0.484 |
| Fear Ratings | CS+ | ranked | post-pre ACQ | pre-post EXT | 0.497 | 0.094 | 0.313 | 0.681 | 5.267 | 90 | 0.000 | 0.202 | 0.253 |
| Fear Ratings | CS+ | ranked | post ACQ | pre-post EXT | 0.442 | 0.090 | 0.266 | 0.618 | 4.930 | 96 | 0.000 | 0.191 | 0.236 |
| Fear Ratings | CS+ | ranked | post-pre ACQ | post EXT | 0.070 | 0.136 | -0.197 | 0.337 | 0.517 | 92 | 0.607 | 0.003 | 0.003 |
| Fear Ratings | CS+ | ranked | post ACQ | post EXT | 0.208 | 0.119 | -0.025 | 0.441 | 1.744 | 101 | 0.084 | 0.029 | 0.030 |
| Fear Ratings | CS+ | ranked | post ACQ | 1st trial RI-Test | 0.364 | 0.087 | 0.193 | 0.535 | 4.192 | 85 | 0.000 | 0.162 | 0.193 |
| Fear Ratings | CS+ | ranked | post-pre ACQ | 1st trial RI-Test | 0.286 | 0.096 | 0.098 | 0.474 | 2.985 | 79 | 0.004 | 0.092 | 0.102 |
| Fear Ratings | CS+ | ranked | pre EXT | 1st trial RI-Test | 0.382 | 0.081 | 0.223 | 0.541 | 4.732 | 82 | 0.000 | 0.198 | 0.247 |
| Fear Ratings | CS+ | ranked | pre-post EXT | 1st trial RI-Test | 0.228 | 0.089 | 0.054 | 0.402 | 2.568 | 81 | 0.012 | 0.066 | 0.071 |
| Fear Ratings | CS+ | ranked | post EXT | 1st trial RI-Test | 0.166 | 0.076 | 0.017 | 0.315 | 2.199 | 84 | 0.031 | 0.056 | 0.059 |
| Fear Ratings | CS- | ranked | post-pre ACQ | pre EXT | 0.430 | 0.169 | 0.099 | 0.761 | 2.552 | 86 | 0.012 | 0.090 | 0.098 |
| Fear Ratings | CS- | ranked | post ACQ | pre EXT | 0.558 | 0.093 | 0.376 | 0.740 | 5.965 | 98 | 0.000 | 0.276 | 0.381 |
| Fear Ratings | CS- | ranked | post-pre ACQ | pre-post EXT | 0.086 | 0.136 | -0.181 | 0.353 | 0.629 | 85 | 0.531 | 0.006 | 0.006 |
| Fear Ratings | CS- | ranked | post ACQ | pre-post EXT | 0.192 | 0.080 | 0.035 | 0.349 | 2.405 | 97 | 0.018 | 0.059 | 0.062 |
| Fear Ratings | CS- | ranked | post-pre ACQ | post EXT | 0.250 | 0.167 | -0.077 | 0.577 | 1.500 | 88 | 0.137 | 0.030 | 0.031 |
| Fear Ratings | CS- | ranked | post ACQ | post EXT | 0.443 | 0.098 | 0.251 | 0.635 | 4.512 | 100 | 0.000 | 0.171 | 0.206 |
| Fear Ratings | CS- | ranked | post ACQ | 1st trial RI-Test | 0.144 | 0.081 | -0.015 | 0.303 | 1.775 | 88 | 0.079 | 0.037 | 0.038 |
| Fear Ratings | CS- | ranked | post-pre ACQ | 1st trial RI-Test | 0.050 | 0.123 | -0.191 | 0.291 | 0.411 | 78 | 0.682 | 0.002 | 0.002 |
| Fear Ratings | CS- | ranked | pre EXT | 1st trial RI-Test | 0.148 | 0.075 | 0.001 | 0.295 | 1.979 | 86 | 0.051 | 0.043 | 0.045 |
| Fear Ratings | CS- | ranked | pre-post EXT | 1st trial RI-Test | 0.003 | 0.103 | -0.199 | 0.205 | 0.025 | 85 | 0.980 | 0.000 | 0.000 |
| Fear Ratings | CS- | ranked | post EXT | 1st trial RI-Test | 0.249 | 0.071 | 0.110 | 0.388 | 3.503 | 87 | 0.001 | 0.126 | 0.145 |
| *Note*. Stim. = Stimulus, CI = Confidence Interval, CS dis. = CS discrimination, pre = prior to the experimental phase, post = subsequent to the experimental phase, ACQ = Acquisition training, EXT = Extinction training, RI = Reinstatement, RI-Test = Reinstatement-Test. | | | | | | | | | | | | | |

**References**

Cohen, J. (1988). *Statistical power analysis for the behavioral sciences* (2nd ed). L. Erlbaum Associates.

Selya, A. S., Rose, J. S., Dierker, L. C., Hedeker, D., & Mermelstein, R. J. (2012). A Practical Guide to Calculating Cohen’s f2, a Measure of Local Effect Size, from PROC MIXED. *Frontiers in Psychology*, *3*. <https://doi.org/10.3389/fpsyg.2012.00111>
